# IL-17C and IL-17RE Promote Wound Closure in a *Staphylococcus aureus*-Based Murine Wound Infection Model

**DOI:** 10.3390/microorganisms9091821

**Published:** 2021-08-27

**Authors:** Linda Pätzold, Alexandra Stark, Felix Ritzmann, Carola Meier, Thomas Tschernig, Jörg Reichrath, Robert Bals, Markus Bischoff, Christoph Beisswenger

**Affiliations:** 1 Institute for Medical Microbiology and Hygiene, Saarland University, 66421 Homburg, Germany; linda.paetzold@uks.eu; 2 Department of Dermatology, Saarland University Hospital, 66421 Homburg, Germany; Alexandra.Stark@uks.eu (A.S.); Joerg.Reichrath@uks.eu (J.R.); 3 Department of Internal Medicine V—Pulmonology, Allergology and Critical Care Medicine, Saarland University, 66421 Homburg, Germany; Felix.Ritzmann@uks.eu (F.R.); Robert.Bals@uks.eu (R.B.); 4 Institute of Anatomy and Cell Biology, Saarland University, 66421 Homburg, Germany; Carola.Meier@uks.eu (C.M.); Thomas.Tschernig@uks.eu (T.T.)

**Keywords:** *Staphylococcus aureus*, wound infection, interleukin-17C, wound closure

## Abstract

The epithelial cytokine interleukin-17C (IL-17C) mediates inflammation through the interleukin 17 receptor E (IL-17RE). Prior studies showed a detrimental role of IL-17C in the pathogenesis of immune-mediated skin diseases (e.g., psoriasis). Here, we examined the role of IL-17C/IL-17RE in wound closure in a *Staphylococcus aureus* wound infection model. We demonstrate that wound closure is significantly delayed in IL-17RE (*Il-17re^−/−^*)- and 17C (*Il-17c^−/−^*)-deficient mice. There was no significant difference between WT, *Il-17re^−/−^*, and *Il-17c^−/−^* mice in the absence of infection. Deficiency for IL-17RE and IL-17C did not significantly affect the elimination of bacteria. IL-17C expression was increased in the epidermis of human *S. aureus*-infected skin. Our results indicate that the IL-17C/IL-17RE axis contributes to the closure of infected wounds but does not contribute to the elimination of *S. aureus*.

## 1. Introduction

The interleukin 17 (IL-17) family comprises six subtypes (IL-17 A to F) which bind to homo- and hetero-dimeric receptor complexes of the IL-17 receptor family (IL-17RA to IL-RE) [1]. IL-17C is mainly expressed by epithelial cells and mediates the expression of cytokines, chemokines, and antimicrobial peptides through a receptor complex of IL-17RA and IL-17RE, NF-kB, and MAP kinases [2,3,4]. Inflammatory mediators (e.g., TNF-α, IL-1β) induce the expression of IL-17C in keratinocytes [2,3,5,6,7,8,9]. Thus, a variety of studies have examined the function of IL-17C in the context of psoriasis and atopic dermatitis. Johnston et al. [8] showed that the overexpression of IL-17C in keratinocytes resulted in an increased proliferation of keratinocytes and the development of a psoriasiform-skin phenotype in mice. Ramirez-Carrozzi et al. [3] demonstrated that IL-17C promotes imiquimod-dependent skin-inflammation, epidermal thickening, and keratinocyte proliferation. In addition, studies have demonstrated that IL-17C is expressed in the skin of patients with atopic dermatitis, and that the treatment with therapeutic antibodies directed against IL-17C decreased skin inflammation in IL-23- and calcipotriol-dependent mouse models of psoriasis and atopic dermatitis, respectively [5,6,8].

Bacterial infections of wounds are a major health issue worldwide and cause considerable costs to public health care systems [10]. A commonly found bacterial species in chronic wound infection is *Staphylococcus aureus*, which delays wound closure and causes pain to the patient [10]. Notably, Roth et al. [11] showed that the pattern recognition receptor NOD2 mediates *S. aureus*-induced IL-17C expression in keratinocytes. Given that IL-17C mediates keratinocyte proliferation and epidermal thickening in experimental models of psoriasis and atopic dermatitis, we wondered about the function(s) of the IL-17C/IL-17RE axis in the healing of wounds infected with *S. aureus*. Here, we studied the function of the IL-17C/IL-17RE axis in a murine *S. aureus* wound infection model and determined the expression of IL-17C in human *S. aureus*-infected skin.

## 2. Materials and Methods

### 2.1. S. aureus-Based Murine Wound Infection Model

Animal experiments were performed with approval of the local State Review Boards of Saarland, Germany (project identification codes 41/2017, approved 19 January 2018, and 30/2018, approved 2 August 2018) and were conducted following the national and European guidelines for the ethical and human treatment of animals. Wounds were introduced in the flanks of female 8- to 12-week-old wild-type (WT), IL-17C-deficient (*Il-17c^−/−^* [12]), and IL-17RE-deficient (*Il-17re^−/−^* [13]) mice (all C57BL/6 background) as described before [14]. In brief, anesthetized mice were treated with carprofen (5 mg/kg bodyweight; Zoetis, Berlin, Germany) and both flanks were shaved and depilated with asid-med hair removal cream (Asid Bonz, Herrenberg, Germany). Skin was disinfected and one full-thickness wound was created on each side with a sterile 5 mm biopsy punch (pfm medical, Köln, Germany). Wounds were stabilized with silicon O-rings (external diameter of 5.5 mm; HUG, Ergolding, Germany) until day 2 to prevent wound healing by contraction and were inoculated with 1 × 10^5^ colony-forming units (CFU) of *S. aureus* strain Newman [15] or phosphate-buffered saline (PBS) without bacteria (control) directly after wounding. The bacterial inoculum was prepared by diluting bacteria from overnight cultures in tryptic soy broth (TSB; BD, Heidelberg, Germany) to an optical density at 600 nm (OD_600_) of 0.05, and incubating the cultures at 37 °C and 225 rpm with a flask-to-medium ratio of 10:1. After 2–2.5 h of growth, cells were harvested by centrifugation, washed twice with PBS, and resuspended in PBS to an OD_600_ of 0.1 (∼1 × 10^7^ CFU/mL). A 10 µl aliquot of the bacterial solution was spotted onto the wound and allowed to infiltrate into the wound bed for 5 min. Afterwards, wounds were covered with air-permeable Tegaderm films (3M, Neuss, Germany), and animals were monitored daily. At day 6 post-infection, wounds were documented, the mice sacrificed, and the wound areas excised and homogenized in PBS with a hand dispenser (Polytron PT 1200 E Kinematica, Eschbach, Germany). Numbers of CFU were determined by plating serial dilutions on sheep blood agar.

### 2.2. Cytokine Determinations in Wound Tissue Homogenates

Wound homogenates obtained from excised wound areas of *S. aureus*-infected WT-, *Il-17c^−/−^*, and *Il-17re^−/−^* mice at day 6 post-infection were centrifugated at 4 °C and 2500× *g* for 10 min. Concentrations of cytokines were measured in supernatants using ELISAs for G-CSF, IL-6, KC, MIP-2, and TNF-α (R&D Systems, Minneapolis, MN, USA) as described by the manufacturer.

### 2.3. Histologic and Immunohistochemical Analyses of Infected Wounds

On day 6 post-infection, mice were euthanized, the entire wound areas excised and fixed overnight in 4% buffered paraformaldehyde at 4 °C, followed by dehydration and embedding of the samples in paraffin. Skin sections were cut into 5 µm in thickness, and placed on slides. Hematoxylin and eosin (H&E)-stained slides were prepared using routine methods. Ki-67 was stained with the primary antibody ab15580, and CD68 with the primary antibody ab125212 (Abcam, Cambridge, UK). Corresponding HRP-conjugated secondary antibodies (Histofine Simple Stain, Nichirei Biosciences Inc., Tokyo, Japan) were used as described in [16]. Keratin 5 (K5) was stained with the primary antibody ab64081 (Abcam), followed by the HRP-conjugated secondary antibody inv10547 (Invitrogen, Karlsruhe, Germany). Photomicrographs of H&E-, CD68- and K5-stained wound sections were taken with a Leica DM100 system (Leica, Wetzlar, Germany) and a MikroCam SP 5.1 microscope camera (Bresser, Rhede, Germany) at 4 to 40-fold magnification. Polymorphonuclear neutrophil (PMN) and macrophage (CD68) numbers in the dermal regions of the infected wounds edges were determined in two randomly chosen high-power fields per slide blinded to the investigator and calculated to numbers/mm^2^. K5 staining intensities of the keratinocytes in the epidermis of infected wound edges were scored (2 = intense staining; 1 = medium staining; 0 = weak to no staining) and a mean score index was calculated per wound. Images of Ki-67 labeled wound sections were taken with an Olympus BX51 microscope system (Olympus, Shinjuku, Japan) using the software Cell Sense Dimension version 1.5 (Olympus). Ki-67-positive cells were counted in the epidermal regions of infected wound edges. The Ki-67 proliferation index was defined as Ki-67-positive cells per 100 cells.

### 2.4. IL-17C Determinations in S. aureus-Infected Wound Biopsies

*S. aureus*-infected wound biopsies and skin biopsies from uninfected donors that were sampled for clinical purposes were obtained anonymously from the Clinic for Dermatology, Venereology and Allergology, Saarland University Hospital, Homburg, Germany. Subsequent usage of these biopsies for research purposes was approved by the Ethics Committee of the Medical Association of Saarland (code number 178/11, approved 10 October 2011). Immunohistochemical staining for IL-17C in human skin sections was performed as described before [17]. IL-17C staining intensities in the epidermal and dermal regions were determined using the image analysis software program Fiji [18], version 1.53c, and normalized to the intensities of the control stain hematoxylin in the respective regions. To determine the IL-17C staining intensity, several images of representative areas were taken for each patient (1 slide per patient). The mean intensity of the whole documented tissue was measured for each field. The data points represent the average of multiple fields from the same slide.

### 2.5. Statistical Analyses

The statistical significance of changes between groups was determined using the Graph-Pad software package Prism 6.01. Identified *p*-values < 0.05 were considered statistically significant. Comparisons between groups were analyzed by a two-way ANOVA and Holm–Sidak’s post hoc test, a one-way ANOVA and Holm–Sidak’s multiple comparison test, or the unpaired Student’s *t*-test as indicated in the figure legends.

## 3. Results and Discussion

### 3.1. Infection with S. aureus Results in a Delayed Wound Closure in Il-17c^−/−^ and Il-17re^−/−^ Mice

**Figure 1:**
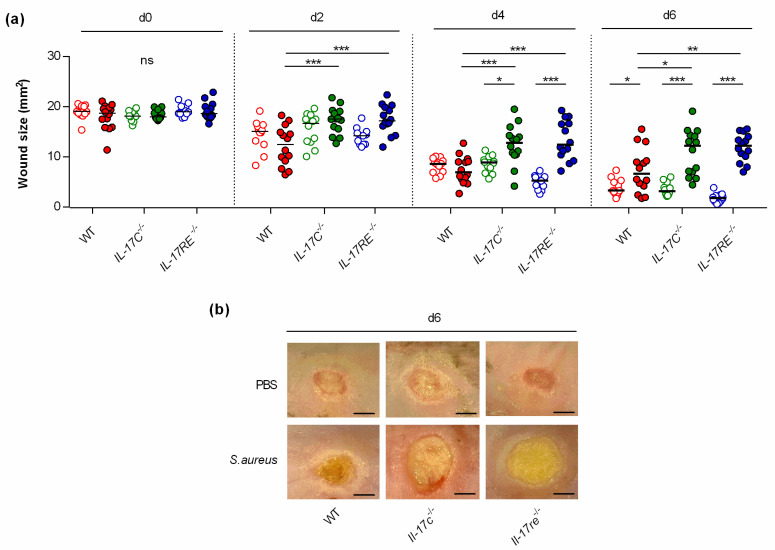
The deficiency for IL-17C and IL-17RE results in a delayed closure of infected wounds. Wounds were introduced into the flanks of WT (red symbols), *Il-17c^−/−^* (green symbols) and *Il-17re^−/−^* (blue symbols) mice and infected with 10^5^ CFU of *S. aureus* strain Newman (filled symbols) or PBS (open symbols) as control. (**a**) The averaged wound areas (mm^2^) are shown 0, 2, 4, and 6 days after wounding (n = 12–14 per group). Each symbol represents an individual wound. Horizontal bars indicate the median of all observations. Data were compared by a two-way ANOVA and Holm–Sidak’s post hoc test. * *p* < 0.05; ** *p* < 0.01; *** *p* < 0.001; ns, not significant. (**b**) Representative images of wounds 6 days after wounding. Scale bar, 2 mm.

To study the function of the IL-17C/IL-17RE axis in wound healing, punch wounds were introduced on both flanks of WT-, *Il-17c^−/−^*, and *Il-17re^−/−^* mice, respectively, either infected with approximately 10^5^ CFU of *S. aureus* strain Newman or treated with an equal volume of PBS as sham-infected control, and wound closure was monitored after day 2, 4, and 6 post-infection (Figure 1). This analysis revealed no clear difference in the wound closure rates between sham-infected WT-, *Il-17c^−/−^*, and *Il-17re^−/−^* mice after 6 days of infection. However, a significant decrease in wound closure was observed in the *S. aureus* Newman-infected WT mice at day 6 post-infection (Figure 1a,b), in line with earlier observations [14]. Notably, the average wound sizes at 2, 4 and 6 days after wounding were significantly increased in infected *Il-17c^−/−^* and *Il-17re^−/−^* mice when compared with those of infected WT mice (Figure 1a), suggesting that the IL-17C/IL-17-RE axis is negligible for the wound healing of uninfected wounds but is important for the wound healing capacity of *S. aureus*-infected wounds.

### 3.2. The Deficiency for IL-17C and IL-17RE Neither Affects Bacterial Numbers in S. aureus-Infected Wounds nor the Recruitment of Polymorphonuclear Neutrophils

**Figure 2:**
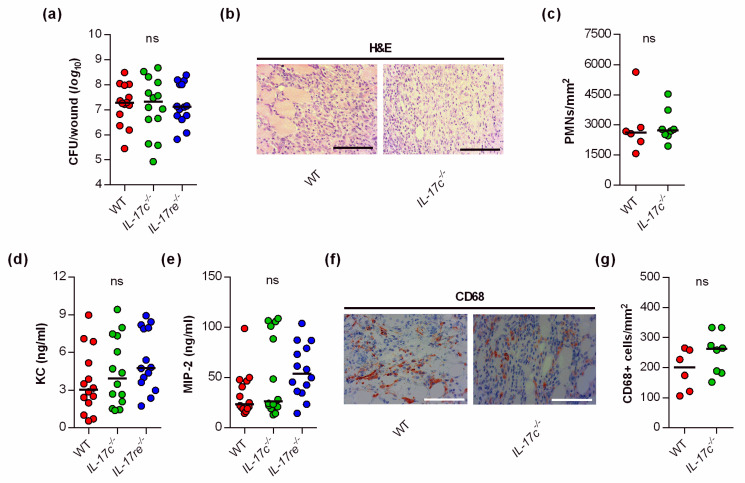
The deficiency for IL-17C and IL-17RE does not affect the bacterial load and PMN content of *S. aureus*-infected wounds. (**a**) Numbers of viable bacteria (CFU) in wounds of WT (red symbols), *Il-17c^−/−^* (green symbols) and *Il-17re^−/−^* (blue symbols) mice at 6 days post-infection. (**b**) Representative images of hematoxylin–eosin (H&E)-stained tissue sections obtained from *S. aureus*-infected wounds of wild-type and *IL-17c^−/−^* mice at 6 days post-infection. Scale bar, 100 µm. (**c**) Numbers of PMNs in the dermal regions of the infected wounds (*n* = 6–8 per group). (**d**,**e**) Concentrations of KC (**d**) and MIP-2 (**e**) in wound homogenates of 6-day-old *S. aureus*-infected wounds (*n* = 14 per group). (**f**) Representative images of CD68-stained tissue sections obtained from *S. aureus*-infected wounds of wild-type and *IL-17c^−/−^* mice at 6 days post-infection. Scale bar, 100 µm. (**g**) Numbers of CD68^+^ cells in the dermal regions of the infected wounds (*n* = 6–8 per group). Each symbol represents an individual wound. Horizontal bars indicate the median of all observations. Data were compared by a one-way ANOVA and Holm–Sidak’s multiple comparison test (**a**,**d**,**e**) or the unpaired Student’s *t*-test (**c**). ns, not significant.

Given the significant impact of the IL-17C and IL-17RE deficiency on the wound healing kinetics of *S. aureus*-infected wounds, we wondered whether and how the IL-17C/IL-17RE axis affects the bacterial burden and immune cell recruitment in the infected tissue. Thus, we next determined the bacterial loads in wound tissues obtained from *S. aureus*-infected WT, *Il-17c^−/−^* and *Il-17re^−/−^* mice at day 6 post-infection (Figure 2a).

Surprisingly, neither the lack of IL-17C nor IL-17RE markedly affected the bacterial loads in the wounds at the end of the experiment (i.e., 6 days post-infection). There was also no significant difference in the bacterial loads between WT and *Il-17re^−/−^* mice 2 days post-infection (data not shown). Similarly, we did not observe clear differences in PMN contents in the infected wound tissues of *S. aureus*-infected WT and *Il-17c^−/−^* mice at day 6 post-infection (Figure 2b,c). In line with the latter observation, we also failed to detect clear differences in keratinocyte-derived chemokine (KC, CXCL1) and macrophage inflammatory protein 2 (MIP-2, CXCL2) contents in wound homogenates of *S. aureus*-infected WT, *Il-17c^−/−^*, and *Il-17re^−/−^* mice at day 6 post-infection (Figure 2d,e), two major players in neutrophil recruitment to infected tissues [19], although concentrations of MIP-2 were about twice as high in *Il-17re^−/−^* mice than in WT mice (Figure 2e; *p* = 0.148 [one-way ANOVA and Holm–Sidak’s multiple comparison test]).

To test whether the IL-17C/IL-17RE axis might affect the influx of macrophages/monocytes into the infected wound areas, we additionally stained wound sections of *S. aureus*-infected wild-type and *Il-17c^−/−^* mice for CD68, a marker for mononuclear phagocytes [20]. Numbers of CD68 positive cells were slightly, but not significantly increased in wounds of *Il-17c^−/−^* mice at day 6 post-infection (Figure 2f,g). Taken together, these findings suggest that the IL-17C/IL-17RE axis seems to play neither a major role in the elimination of the bacterial load nor in the recruitment of PMNs/macrophages to the infected wound tissue, at least during the later stage of infection.

### 3.3. The Deficiency for IL-17C and IL-17RE Only Marginally Affects the Contents of Wound-Healing-Associated Cytokines and Cell Proliferation in S. aureus-Infected Wounds

**Figure 3:**
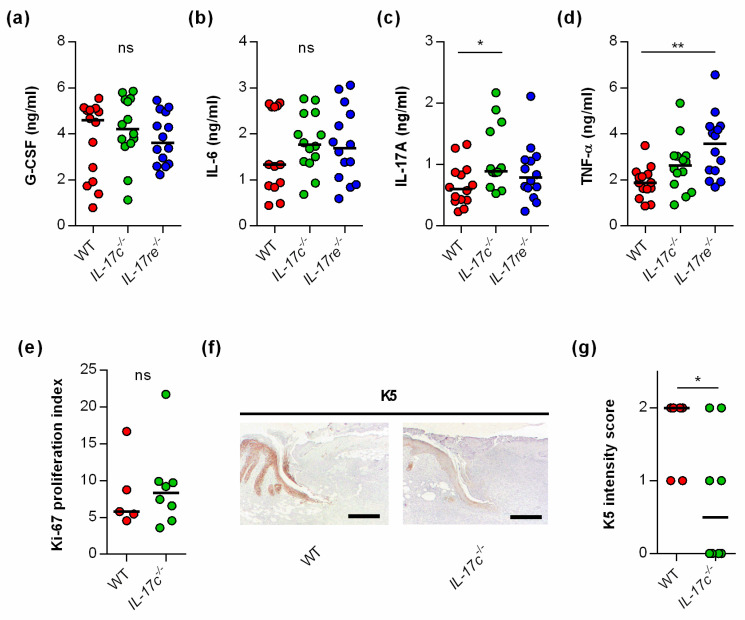
The deficiency for IL-17C and IL-17RE does only marginally affect the contents of wound-healing-associated cytokines in *S. aureus*-infected wounds. (**a**–**d**) Concentrations of G-CSF (**a**), IL-6 (**b**), IL-17A (**c**), and TNF-α (**d**) in wound homogenates of 6 days old *S. aureus*-infected wounds (*n* = 14 per group). (**e**) Ki-67 proliferation indices of tissue sections obtained from *S. aureus*-infected wounds of wild-type and *IL-17c^−/−^* mice at 6 days post-infection (*n* = 5–7). (**f**) Representative images of K5-stained tissue sections obtained from *S. aureus*-infected wounds of WT and *IL-17c^−/−^* mice at 6 days post-infection. Scale bar, 1 mm. (**g**) Intensity scores of K5-positive cells in the epidermal regions of the infected wound edges (*n* = 6–8 per group). Each symbol represents an individual wound. Horizontal bars indicate the median of all observations. Data were compared by one-way ANOVA and Holm–Sidak’s multiple comparison test (**a**–**d**) or the unpaired Student’s *t*-test (**e**,**g**). ns, not significant; * *p* < 0.05; ** *p* < 0.01.

To test whether the IL-17C/IL-17RE axis might affect tissue repair of *S. aureus*-infected wounds via the modulation of other wound-healing promoting/decelerating cytokines, we next determined the concentrations of granulocyte colony-stimulating factor (G-CSF) [21], interleukin-6 (IL-6) [22], interleukin-17A (IL-17A) [23], and tumor necrosis factor α (TNF-α) [24] in wound homogenates (Figure 3).

No clear differences in cytokine levels were observed in wound homogenates of *S. aureus*-infected WT, *Il-17c^−/−^*, and *Il-17re^−/−^* mice for the wound-closure-accelerating cytokines G-CSF and IL-6 [21,22] (Figure 3a,b). However, significant differences in cytokine expression between *S. aureus*-infected WT, *Il-17c^−/−^* and *Il-17re^−/−^* mice were found for the skin wound-healing-impairing cytokines IL-17A and TNF-α [23,24] at day 6 post-infection, which were significantly increased in wound homogenates obtained from *Il-17c^−/−^* mice and *Il-17re^−/−^* mice, respectively (Figure 3c,d).

To test whether the IL-17C/IL-17RE axis might promote wound closure of *S. aureus*-infected wounds by stimulating cell proliferation or keratinocyte differentiation, we also determined the Ki-67 and keratin 5 (K5) contents in wound edges of *S. aureus*-infected WT and *Il-17c^−/−^* mice at day 6 post-infection (Figure 3e–g). Although no clear difference in the Ki-67 proliferative indices between the wounds of *S. aureus*-infected WT and *Il-17c^−/−^* mice were identified (Figure 3e), we observed a higher K5 staining intensity in the epidermal regions of *S. aureus*-infected wound edges obtained from wild-type mice (Figure 3f,g).

Taken together, these data suggest that the IL-17C/IL-17RE axis might promote the wound closure of *S. aureus*-infected wounds via the modulation of IL17A/TNF-α expression and/or the formation of K5/K14 intermediate filament-producing keratinocytes at the wound edges but does not markedly alter cell proliferation in *S. aureus*-infected wounds.

### 3.4. IL-17C Is Expressed in S. aureus-Infected Human Skin

**Figure 4:**
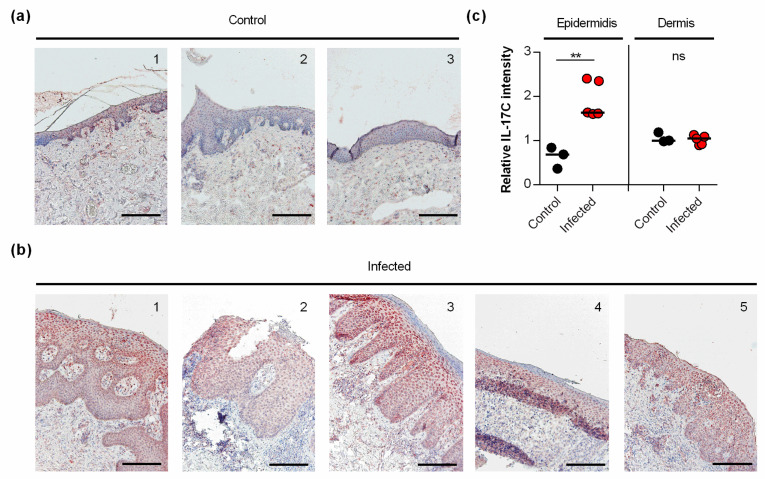
IL-17C is expressed in *S. aureus*-infected skin (**a**,**b**). IL-17C was detected by immunohistochemistry in formalin-fixed, paraffin-embedded skin samples obtained from 3 non-infected individuals (**a**) and 5 patients with *S. aureus* skin infection (**b**). Scale bar, 100 µm. (**c**) The relative IL-17C intensity was quantified in the epidermis and dermis in relation to the staining intensities seen with hematoxylin in the respective regions. Relative IL-17C intensities between non-infected (black symbols) and *S. aureus* infected (red symbols) wound biopsies were compared by unpaired Student’s *t*-test. **, *p* < 0.01.

Earlier work demonstrated that *S. aureus* induces the transcription of IL-17C in keratinocytes via NOD2 activation [11]. However, to the best of our knowledge, it remains unknown whether and how *S. aureus* affects the expression of IL-17C in infected skin. Thus, we also evaluated whether and how IL-17C is expressed in *S. aureus*-infected human skin (Figure 4). We found strong expression of IL-17C in tissue slices of *S. aureus*-infected skin biopsies, particularly in the granular and spinous layers of the harmed epidermidis (i.e., stratum granulosum and stratum spinosum; Figure 4a). Semi-quantitative evaluation of the IL-17C staining showed that the expression of IL-17C was significantly increased in the epidermis of *S. aureus*-infected skin biopsies compared to non-infected control biopsies, whereas no clear differences in IL-17C staining were observed in the dermal regions of *S. aureus*-infected and non-infected biopsies (Figure 4b), suggesting that IL-17C production is increased, particularly in the epidermal regions of *S. aureus*-infected wound but not in the lower parts of the infected skin.

## 4. Conclusions

Here, we demonstrate that the IL-17C/IL-17RE axis promotes wound closure in a *S. aureus* wound infection model; however, it does not affect immune cell recruitment, bacterial clearance, or the expression of wound-healing-associated cytokines. Additionally, we could not identify any function for IL-17C and IL-17RE in the absence of infection. As the expression of IL-17C depends on microbial factors and inflammatory mediators, the latter result was to be expected. We further demonstrated that the expression of IL-17C is increased in the epidermis of *S. aureus*-infected human skin. Increased expression of IL-17C could also be detected in psoriasis and atopic dermatitis [6,8]. Thus, epidermal expression of IL-17C seems to be a common feature of inflamed skin. Contrary to our wound model, pre-clinical studies showed a detrimental role of the IL-17C/IL-17RE axis in the pathogenesis of immune-mediated skin diseases. Different mouse models of psoriasis and atopic dermatitis demonstrated that IL-17C mediates skin inflammation, epidermal thickening, and keratinocyte proliferation [3,5,6,8]. Our finding that wound healing is delayed in infected wounds in the absence of IL-17C and IL-17RE is in line with the mentioned studies. In our wound-healing model, as well as in the models of experimental psoriasis and in lung cancer models [3,8,16], IL-17C mediates cell proliferation and growth. Thus, IL-17C/IL-17RE contributes to wound healing in infected wounds, but is detrimental in immune-mediated diseases, such as psoriasis. Moreover, *S. aureus* is frequently found in the skin of patients with psoriasis and atopic dermatitis [25,26]. Therefore, it is tempting to speculate whether *S. aureus* contributes to the expression of IL-17C in psoriasis and atopic dermatitis and, thereby, to the IL-17C-mediated disease progression. The elimination of an aberrant microbiota often found in these chronic skin diseases [27] could result in a reduced IL-17C-mediated skin inflammation and keratinocyte proliferation.

Our study has limitations. We did not analyze, in depth, the cellular mechanisms underlying IL-17C/IL-RE-mediated healing of infected wounds. As delayed wound closure in IL-17C- and IL-17RE-deficient mice was already visible at 2 days post-infection, IL-17C/IL-17RE-mediated cell proliferation and differentiation is likely an early event in our model. Roth et al. [11] showed that viable *S. aureus* induced IL-17C expression in keratinocytes within 6 h post-infection. Thus, it is possible that IL-17C accelerates wound closure by supporting keratinocyte survival and the differentiation of wounds shortly after infection without affecting the clearance of *S. aureus*. In the later course of infection, IL-17A (which shares the receptor IL-17RA with IL-17C) released from immune cells such as γδ T cell may compensate the loss of IL-17C-signaling in *S. aureus*-infected wounds [28,29]. This may explain why we could not detect a clear difference in the proliferation index at 6 days post-infection when the wound lesions were embedded, although an increased expression of K5 was noticed in keratinocytes at the wound edges of *S. aureus*-infected WT mice. The molecular mechanism(s) modulated by IL-17C/IL-17RE to promote K5 expression and wound closure of *S. aureus*-infected wounds still needs to be identified.

## Data Availability

The datasets used and/or analyzed during the current study are available from the corresponding author on reasonable request.

## References

[1] Monin L., Gaffen S.L. (2018). Interleukin 17 Family Cytokines: Signaling Mechanisms, Biological Activities, and Therapeutic Implications. Cold Spring Harb. Perspect. Biol..

[2] Song X., Zhu S., Shi P., Liu Y., Shi Y., Levin S.D., Qian Y. (2011). IL-17RE is the functional receptor for IL-17C and mediates mucosal immunity to infection with intestinal pathogens. Nat. Immunol..

[3] Ramirez-Carrozzi V., Sambandam A., Luis E., Lin Z., Jeet S., Lesch J., Hackney J., Kim J., Zhou M., Lai J. (2011). IL-17C regulates the innate immune function of epithelial cells in an autocrine manner. Nat. Immunol..

[4] Kusagaya H., Fujisawa T., Yamanaka K., Mori K., Hashimoto D., Enomoto N., Inui N., Nakamura Y., Wu R., Maekawa M. (2014). Toll-like receptor-mediated airway IL-17C enhances epithelial host defense in an autocrine/paracrine manner. Am. J. Respir. Cell Mol. Biol..

[5] Lauffer F., Jargosch M., Baghin V., Krause L., Kempf W., Absmaier-Kijak M., Morelli M., Madonna S., Marsais F., Lepescheux L. (2020). IL-17C amplifies epithelial inflammation in human psoriasis and atopic eczema. J. Eur. Acad. Derm. Venereol..

[6] Vandeghinste N., Klattig J., Jagerschmidt C., Lavazais S., Marsais F., Haas J.D., Auberval M., Lauffer F., Moran T., Ongenaert M. (2018). Neutralization of IL-17C Reduces Skin Inflammation in Mouse Models of Psoriasis and Atopic Dermatitis. J. Investig. Derm..

[7] Peng T., Chanthaphavong R.S., Sun S., Trigilio J.A., Phasouk K., Jin L., Layton E.D., Li A.Z., Correnti C.E., De van der Schueren W. (2017). Keratinocytes produce IL-17c to protect peripheral nervous systems during human HSV-2 reactivation. J. Exp. Med..

[8] Johnston A., Fritz Y., Dawes S.M., Diaconu D., Al-Attar P.M., Guzman A.M., Chen C.S., Fu W., Gudjonsson J.E., McCormick T.S. (2013). Keratinocyte overexpression of IL-17C promotes psoriasiform skin inflammation. J. Immunol..

[9] Johansen C., Riis J.L., Gedebjerg A., Kragballe K., Iversen L. (2011). Tumor necrosis factor alpha-mediated induction of interleukin 17C in human keratinocytes is controlled by nuclear factor kappaB. J. Biol. Chem..

[10] Tong S.Y., Davis J.S., Eichenberger E., Holland T.L., Fowler V.G. (2015). Staphylococcus aureus infections: Epidemiology, pathophysiology, clinical manifestations, and management. Clin. Microbiol. Rev..

[11] Roth S.A., Simanski M., Rademacher F., Schroder L., Harder J. (2014). The pattern recognition receptor NOD2 mediates Staphylococcus aureus-induced IL-17C expression in keratinocytes. J. Investig. Derm..

[12] Jungnickel C., Schmidt L.H., Bittigkoffer L., Wolf L., Wolf A., Ritzmann F., Kamyschnikow A., Herr C., Menger M.D., Spieker T. (2017). IL-17C mediates the recruitment of tumor-associated neutrophils and lung tumor growth. Oncogene.

[13] Steck P., Ritzmann F., Honecker A., Vella G., Herr C., Gaupp R., Bischoff M., Speer T., Tschernig T., Bals R. (2019). Interleukin 17 Receptor E (IL-17RE) and IL-17C Mediate the Recruitment of Neutrophils during Acute Streptococcus pneumoniae Pneumonia. Infect. Immun..

[14] Haupenthal J., Kautz Y., Elgaher W.A.M., Pätzold L., Röhrig T., Laschke M.W., Tschernig T., Hirsch A.K.H., Molodtsov V., Murakami K.S. (2020). Evaluation of Bacterial RNA Polymerase Inhibitors in A. ACS Infect. Dis..

[15] Bae T., Baba T., Hiramatsu K., Schneewind O. (2006). Prophages of Staphylococcus aureus Newman and their contribution to virulence. Mol. Microbiol..

[16] Ritzmann F., Jungnickel C., Vella G., Kamyschnikow A., Herr C., Li D., Menger M.M., Angenendt A., Hoth M., Lis A. (2019). IL-17C-mediated innate inflammation decreases the response to PD-1 blockade in a model of Kras-driven lung cancer. Sci. Rep..

[17] Pfeifer P., Voss M., Wonnenberg B., Hellberg J., Seiler F., Lepper P.M., Bischoff M., Langer F., Schafers H.J., Menger M.D. (2013). IL-17C is a mediator of respiratory epithelial innate immune response. Am. J. Respir. Cell Mol. Biol..

[18] Schindelin J., Arganda-Carreras I., Frise E., Kaynig V., Longair M., Pietzsch T., Preibisch S., Rueden C., Saalfeld S., Schmid B. (2012). Fiji: An open-source platform for biological-image analysis. Nat. Methods.

[19] Lentini G., Famà A., Biondo C., Mohammadi N., Galbo R., Mancuso G., Iannello D., Zummo S., Giardina M., De Gaetano G.V. (2020). Neutrophils Enhance Their Own Influx to Sites of Bacterial Infection via Endosomal TLR-Dependent Cxcl2 Production. J. Immunol..

[20] Holness C.L., Simmons D.L. (1993). Molecular cloning of CD68, a human macrophage marker related to lysosomal glycoproteins. Blood.

[21] Huang H., Zhang Q., Liu J., Hao H., Jiang C., Han W. (2017). Granulocyte-Colony Stimulating Factor (G-CSF) Accelerates Wound Healing in Hemorrhagic Shock Rats by Enhancing Angiogenesis and Attenuating Apoptosis. Med Sci. Monit. Int. Med. J. Exp. Clin. Res..

[22] Lin Z.Q., Kondo T., Ishida Y., Takayasu T., Mukaida N. (2003). Essential involvement of IL-6 in the skin wound-healing process as evidenced by delayed wound healing in IL-6-deficient mice. J. Leukoc. Biol..

[23] Takagi N., Kawakami K., Kanno E., Tanno H., Takeda A., Ishii K., Imai Y., Iwakura Y., Tachi M. (2017). IL-17A promotes neutrophilic inflammation and disturbs acute wound healing in skin. Exp. Dermatol..

[24] Ashcroft G.S., Jeong M.J., Ashworth J.J., Hardman M., Jin W., Moutsopoulos N., Wild T., McCartney-Francis N., Sim D., McGrady G. (2012). Tumor necrosis factor-alpha (TNF-α) is a therapeutic target for impaired cutaneous wound healing. Wound Repair Regen..

[25] Iwamoto K., Moriwaki M., Miyake R., Hide M. (2019). Staphylococcus aureus in atopic dermatitis: Strain-specific cell wall proteins and skin immunity. Allergol. Int..

[26] Ng C.Y., Huang Y.H., Chu C.F., Wu T.C., Liu S.H. (2017). Risks for Staphylococcus aureus colonization in patients with psoriasis: A systematic review and meta-analysis. Br. J. Dermatol..

[27] Bjerre R.D., Bandier J., Skov L., Engstrand L., Johansen J.D. (2017). The role of the skin microbiome in atopic dermatitis: A systematic review. Br. J. Dermatol..

[28] Cho J.S., Pietras E.M., Garcia N.C., Ramos R.I., Farzam D.M., Monroe H.R., Magorien J.E., Blauvelt A., Kolls J.K., Cheung A.L. (2010). IL-17 is essential for host defense against cutaneous Staphylococcus aureus infection in mice. J. Clin. Investig..

[29] Marchitto M.C., Dillen C.A., Liu H., Miller R.J., Archer N.K., Ortines R.V., Alphonse M.P., Marusina A.I., Merleev A.A., Wang Y. (2019). Clonal Clonal Vγ6+Vδ4+ T Cells promote IL-17–mediated immunity against Staphylococcus aureus skin infection. Proc. Natl. Acad. Sci. USA.

